# Escape rooms as an interactive learning experience: insights into designing a radiology-themed escape room and exit survey data

**DOI:** 10.1186/s13244-025-02127-x

**Published:** 2025-11-01

**Authors:** Jonas Oppenheimer, Sophia Lüken, Annika Bierbrauer, Paul Kamieniarz, Martine S. Nilssen, Maurice Quang Loc Bui, Anna-Maria Haack, Mona Jahn, Katharina Beller, Yasmin Uluk, Lyel Grumberg, Markus Herbert Lerchbaumer, Timo A. Auer, Carolina Dominguez Aleixo, Laura Segger

**Affiliations:** 1 Department of Radiology, Charité-Universitätsmedizin Berlin, Corporate Member of Freie Universität Berlin and Humboldt-Universität zu Berlin, Berlin, Germany; 2 Department of Radiology, Østfold Hospital, Kalnes, Norway; 3 Department of Radiology, BG Klinik Ludwigshafen, Ludwigshafen, Germany

**Keywords:** Education, Medical, Clinical decision-making, Gamification, Interdisciplinary communication

## Abstract

**Objectives:**

Escape rooms provide an interactive learning experience, combining clinical knowledge with problem-solving and teamwork. A radiology-themed escape room has been organized at the European Congress of Radiology in 2019 and 2023–2025, with over 900 people participating in total. The process of developing a radiology-themed escape room is discussed, and the results of a participant survey are presented.

**Materials and methods:**

The development of a radiology-themed escape room was based on five steps. Initially, an overarching concept was chosen, then multiple puzzle ideas were brainstormed. These were linked together to form a story, and then fully developed with relevant images and materials. Finally, the room was tied together, and a fitting atmosphere was created. Participants in 2025 were asked to complete a survey with questions on their training status, the challenges that they found most difficult, and their thoughts on the activity as a learning tool and for improving teamwork.

**Results:**

Three different concepts of radiology-themed escape rooms were developed for the congresses from 2019 to 2025. The overarching concepts were a polytrauma situation, a thrombectomy for fulminant pulmonary embolism, and a tumor board, respectively. Two hundred ninety people participated in 2025, and 149 completed the exit survey; 66.7% of participants were able to complete the room in time. Enjoyment, learning, and team building were all rated highly by participants.

**Conclusion:**

A development process for designing a radiology-themed escape room is presented. A prior implementation shows an enjoyable and educational experience for radiologists and other medical professionals.

**Critical relevance:**

Insights are given on the development of a radiology-themed escape room, providing a unique interactive learning opportunity for residents that incorporates image interpretation with teamwork and cognitive puzzles, resulting in an enjoyable educational experience.

**Key Points:**

- A step-by-step guide on developing a radiology-themed escape room is presented.
- Radiological escape rooms provide an enjoyable, educational, and team-building experience.
- Interactive learning experiences could play a larger role in modern radiology education.

**Graphical Abstract:**

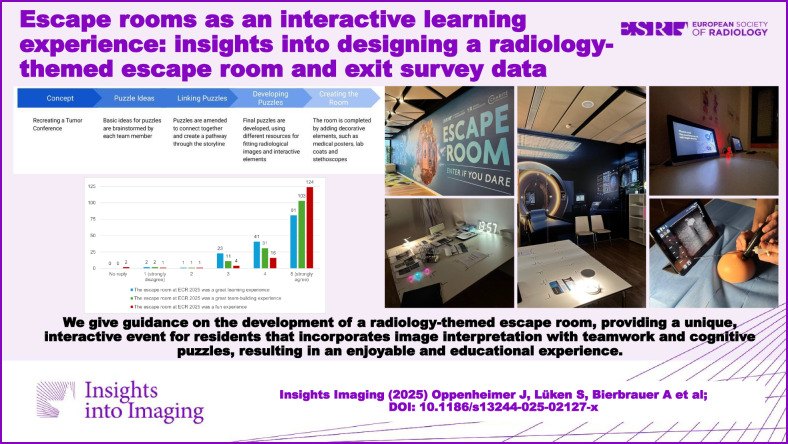

## Introduction

Escape rooms have become a popular pastime since the early 2010s. The concept entails “locking” a group of players into a room with a certain theme and having them solve a series of puzzles to “escape” the room within a set time [1]. The concept of escape rooms has been applied to many different scenarios; while commercial enterprises usually focus on mystery or horror themes, the concept has been adapted to a variety of contexts, including medical scenarios [2]. This type of gamification has been employed as a teaching methodology in several disciplines, demonstrating success in retention of theoretical knowledge [3, 4]. In addition to the cognitively challenging aspect of the puzzles, emphasis is placed on teamwork in order to solve the game in time. Effective communication, leadership, and flexibility have been shown to be crucial in successful completion [5]. Medical-themed escape rooms have been used to improve teamwork and as a didactic method in different aspects of education [6, 7]. Clinical problem-solving situations, in particular, can be reproduced in such rooms, delivering the opportunity to understand complex healthcare structures in a less emergent setting [8]. In a more specific context, radiology-themed escape rooms have previously been described [9].

The team “Young Radiologists e.V.,” consisting of residents and junior consultants from different radiological institutions in several European countries, has been organizing a Summer School for radiology in Berlin, Germany, for medical students since 2018. This led to a cooperation with the European Society of Radiology (ESR), with whom a radiology-themed escape room was developed for the 2019 European Congress of Radiology (ECR) in Vienna, Austria. The team returned to the ECR in 2023 after a COVID-19-related hiatus with a new set of puzzles, with further iterations in 2024 and 2025. Based on the initial concept, a 10-min mini-escape room was developed for the “Young Radiologists e.V.” summer schools [10]. Additionally, the rooms were set up at different national radiology conferences throughout Europe. Overall, more than 900 people participated in the experience. In the latest edition of ECR 2025, participants were asked to fill out an exit survey to gain insights into the impact of escape rooms as a learning tool and a setting for building teamwork. This study aims to give insights into developing a radiology-themed escape room, show the basic concepts of the rooms previously developed, and discuss the results of the survey.

## Materials and methods

### Developing a radiology-themed escape room

**Fig. 1:**
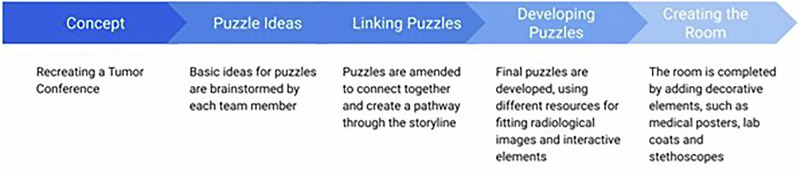
Basic steps of creating a radiology-themed escape room from initial concept to final room setup

Planning of the escape room began six months prior to the ECR. The fundamental steps in planning an escape room are presented in Fig. 1 and detailed below. The local ethics board at the Charité-Universitätsmedizin Berlin, Germany, granted approval for this study (case number: EA2/087/25).

Initially, an overarching concept for the room that provided a through-line for the story, was agreed upon. Clinical challenges encountered daily, such as doing a “FAST” exam (focused assessment with sonography for trauma) in the trauma unit or preparing for a multidisciplinary conference, provided ideas for the concept.

In the next step, each team member brainstormed puzzles for the room, often incorporating their particular interests and clinical experience into the general theme. At this stage, it was not necessary to define the puzzle fully. Ideas ranged broadly from creating literal puzzles from X-rays, coding diagnoses into numbers, and creating a binary code from distinguishing between T1 and T2 weighting in a series of MRI images.

The third step involved incorporating these puzzles into each other to create a playable room. This required multiple meetings to discuss how all puzzles linked together to lead to the completion of the room and the “escape.” Some ideas were discarded, and others were altered to better fit into the game. The venue provided two connected rooms, this division into halves was integrated into gameplay.

In the next step, the puzzles were developed. The team relied on many resources to find images for the puzzles and to realize technical solutions to solve them. The difficulty was adjusted for radiology residents in particular. Old X-ray images were used, as well as external image databases, particularly radiopaedia.org (Radiopaedia Australia Pty Ltd) were relied on, as images are usable for educational purposes with attribution. PowerPoint (Microsoft Corporation) was used to interactively implement puzzles, leading participants through a puzzle with little outside assistance. Haptic elements were purchased from online marketplaces, where different forms of locks, and other useful items, such as ultraviolet (UV) lights, can be found. Medical device manufacturers supplied specialized elements. Mobile devices were incorporated into the rooms, e.g., by enacting a fake phone call via a speaker.

Finally, the experience was deepened by creating a fitting atmosphere. Indirect lighting with low brightness was incorporated, and music was chosen for ambiance. Different decorating elements were used to act as diversions and to create a suitable medical setting.

### Executing the escape room

Each group was allotted 20 min for the room, with a 10-min break in between. Groups were composed of two to five participants. Smaller groups of two to three could be combined to form a full team. Booking of time slots was available online and at a welcome desk on-site.

Three organizing team members were present at all times. Participants were greeted, and the basic concept of an escape room was explained. A short introduction was read to set the scene. Once inside, the timer was started. At least one staff member entered the room with the participants, surveying the group and giving hints when necessary. For each hint given, depending on the extent, a penalty of 30 s or 1 min was added to the final time. After participants entered the second room, the staff would start resetting the puzzles. Once the group solved the final puzzle, the timer was stopped, and the players could “exit”. For safety, the rooms were never actually locked, and participants could leave at any time. Final times were collected, as the ESR provided rewards for the best groups.

### Exit survey

At the ECR 2025, participants were asked to complete a 5-min exit survey to gather data about their country of origin and training status, as well as to give feedback. Exit times for the room were collected in the survey, and by the organizing team, with the inclusion of time penalties for hints; results were rounded to the nearest 30 s. Participants were asked to rate the difficulty and to agree or disagree with the statements that the escape room was a great learning experience, a great team-building experience, and a fun experience. Additionally, participants were asked to designate which part of the escape room concept proved most difficult for them and their general opinion about interactive learning experiences. Ratings were given on a scale of 1 (strongly disagree) to 5 (strongly agree). Minimal edits to write-in answers were made for disambiguation, where appropriate. The entire survey is shown in Appendix 1. The survey was conducted online with Google Forms (Google LLC). Participation was anonymized and not mandatory; incentivization was given in the form of a ticket to the “ECR 2025 Party” (value: 60€) raffled between participants daily.

Ethics approval for this anonymized study was granted by the local ethics review board at the Charité Universitätsmedizin Berlin. The ESR provided financial support for the materials needed for the escape rooms. No further financial support was obtained.

## Results

### Descriptions of the past escape rooms

Three different escape room concepts were developed, with the version in 2023 being based on the initial concept from 2019. The basic settings are described below, leaving exact answers vague, as versions of the rooms may be carried out in the future. Images of the room set-up are shown in Appendix 2.

#### 2019—Polytrauma (1)

The premise was a trauma call on a night shift, where residents are called to the trauma unit to perform initial imaging on a patient. In the room, the participants are confronted with an ultrasound phantom and an ultrasound probe. They perform a FAST scan to find the pathology. Concordantly, multiple pieces of different X-rays are hidden around the room, which must be found, puzzled together, and viewed on a lightbox. These X-rays show two relevant pathologies. There is a piece of paper in the room, where two questions are written down, for which the participants need to write down the right answers, which can be translated into an alphanumeric code. This code allows for opening a box in which a computer mouse is placed. This computer mouse allows scrolling through a cranial CT to find a pathology. All the found pathologies can be found behind a total of nine numbered revision doors affixed to the wall. The combination of the correct pathologies leads to a code that means the escape room was solved.

#### 2023—Polytrauma (2)

The 2023 escape room is a further development of the polytrauma setting from 2019.

The first quizzes with the ultrasound phantom (FAST/ER FAN, Kyoto Kagaku Co., Ltd.) and the FAST scan with the code are the same, except that the alphanumeric code leads to a number to open a lockbox, containing a UV flashlight. The pieces of X-rays are similar to 2019 but show four pathologies, which can be found behind the revision doors, except that the pathologies behind the revision doors at the wall are only visible with the UV flashlight. The numbers of the correct diagnoses form the code to enter the second room. Inside is a workstation, where three cranial CTs are played in succession, showing different intracranial hemorrhages. These need to be correctly identified, and the abbreviations of the diagnoses are translated into a number. This unlocks a lockbox with a computer mouse inside, with which participants can control a second workstation with CT exams showing different infectious entities. The correct diagnosis must be noted on a crossword-style sheet. Once filled out, marked boxes direct the participants to look in a specific direction, where they find a sheet of paper showing a congratulatory message for finishing the room, visualized with the UV flashlight.

#### 2024—Thrombectomy

The participants were given a case file of a critically ill patient to start. Inside the room, different diagnostic tests for the patient can be found; the most fitting chest X-ray needs to be chosen, an ultrasound image of a deep vein thrombosis can be found and correctly diagnosed from nine numbered revision doors, and the Wells score needs to be calculated. The three numbers gleaned from these open a lockbox with a telephone inside, which plays an automatic message once opened, requesting an emergency CT scan. A video is played of a CT showing an apparent pulmonary embolism. The abbreviation of this diagnosis translates to a number, opening the door to the second room. Here, a training model for a pulmonary embolism thrombectomy (proprietary, Inari Medical, Inc.) is found, which must be performed by the participants. This leads to the stabilization of the patient, visualized by a change in their vital parameters on a computer screen. The normalized blood pressure value is a code to open a lockbox with a computer mouse. This allows the players to click through a series of different CTs with a variety of metastases, finally allowing them to choose the most likely primary cancer based on the metastatic pattern. Once the correct diagnosis is selected, the group is presented with a congratulatory message for completing the room.

#### 2025—Tumor conference

For this room, participants were tasked with taking over a tumor conference. They are given four case files and a tablet PC opened to the camera app before entering. Inside, they find a QR code to scan, linking to a social media account where a fictive character has posted an ultrasound image of their liver disease. The concrete hepatic diagnosis allows the participants to unlock a workstation, where they must click through a series of mammography exams, choosing if they show a benign or malignant lesion, giving them a binary code based on each diagnosis. This unlocks a lockbox with a UV flashlight. Simultaneously, there are two images and a diagnosis sheet hidden for each patient in the room. Once found and correctly sorted for each patient, with the help of the UV flashlight, a question asking for a certain date is made visible. This can easily be answered with posters found in the room, the answer is the code to enter the second room. There, a workstation can be found with three different cases where target lesions for biopsies need to be identified to obtain another code, opening a lockbox with a biopsy needle inside, with which an ultrasound-guided biopsy of a breast phantom (US-9, Kyoto Kagaku Co., Ltd.) is performed. Once a lesion is successfully hit, a video is played congratulating the group on finishing the room.

### Participation data 2023 and 2024

Participation data from the initial escape room 2019 was no longer available. In 2023, 44 teams with 186 participants competed. 28 teams (63.6%) were unable to complete the room in time, the fastest resident-only group finished in 14:52 min. In 2024, 58 teams with 268 participants competed, three teams (5.2%) did not finish in time, and the fastest resident-only group finished in 10:09 min.

### Survey results 2025

**Fig. 2:**
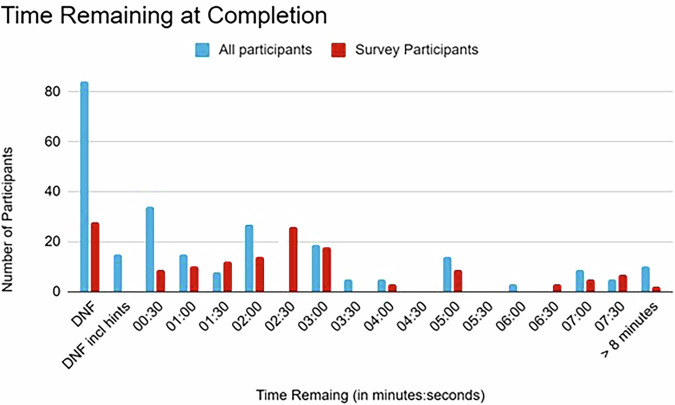
Comparison of the time remaining at completion of the escape room for all participants (blue) and survey participants only (red). A maximum time of 20 min was allotted. DNF, did not finish

In 2025, 63 groups with a total of 290 participants took part in the escape room. Two groups did not show up for their slots and could not be filled on short notice. One additional slot was added on day two. Eighteen groups (28.6%) were unable to finish on time, with a further three groups (4.8%) finishing with a time of over 20 min after the addition of penalties for tips. One hundred forty-nine (51.4%) participants responded to the exit survey. One of these surveys was excluded, as more than three questions were unanswered. Of these, 28 (18.9%) were part of groups that did not finish on time. The fastest resident-only group finished in 12:30 min. A comparison of finishing times is shown in Fig. 2, participants who did not remember their finishing times in the survey were excluded from this analysis.

**Fig. 3:**
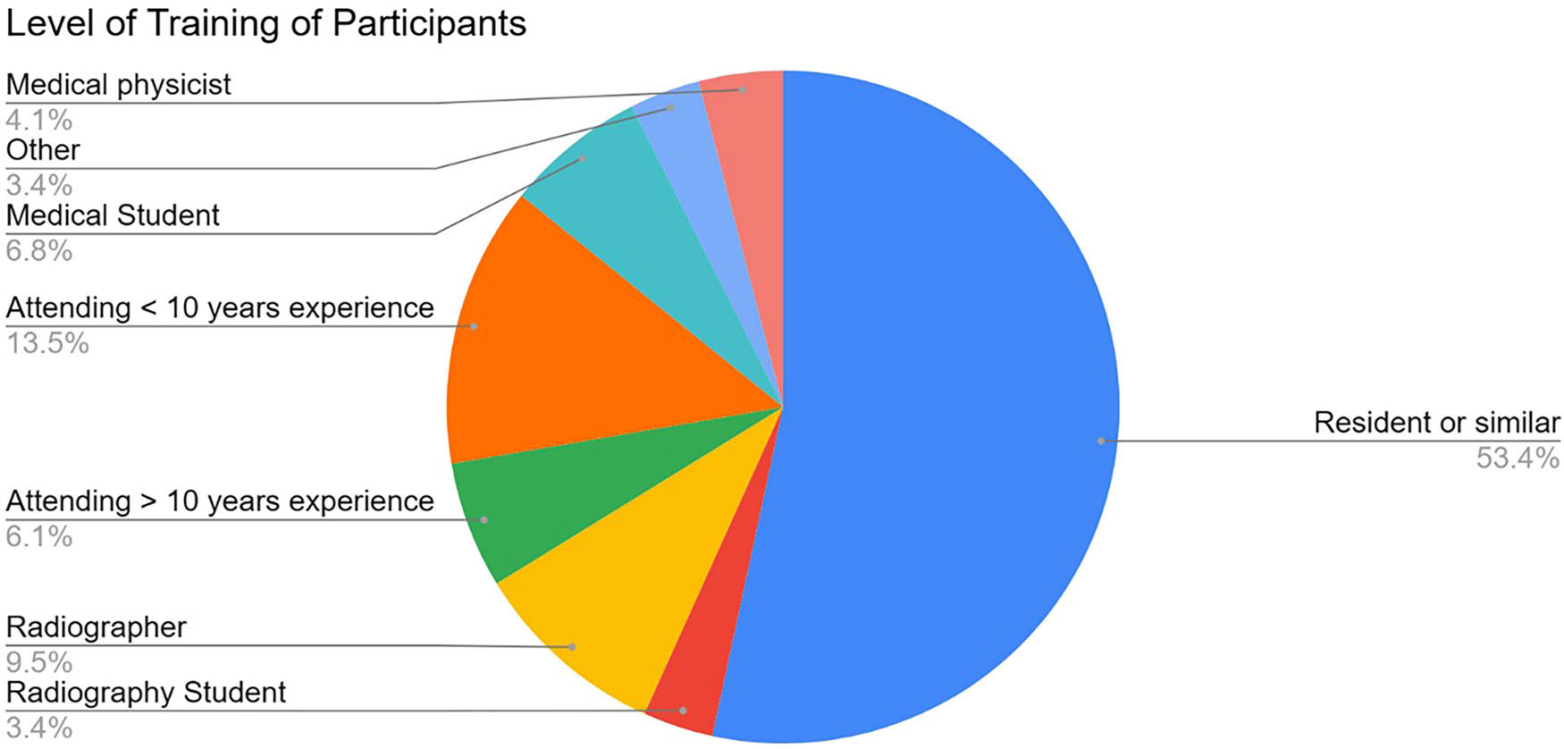
Level of training of the participants in the survey

Participants were from 32 countries of origin, with the largest number from Germany and Romania, at 19 participants each (12.8%). Eleven (7.4%) participants were from non-European countries. The female-to-male ratio was nearly equal (48.6% male, 49.3% female, 2.1% other or no answer). Of the 68.2% of players were in groups of five people, only 2.0% and 3.3% in groups of two or three, respectively. 42.3% of participants were in groups with at least one team member who was non-physician medical staff. Almost half (48.6%) had at least one member who was a board-certified radiologist, while 53.4% of respondents replied that their training status was that of a resident or the equivalent. The breakdown of the training status of participants is shown in Fig. 3. 42.3% of respondents had never played a “regular” escape room previously, and only 20 participants had participated in a previous ECR escape room.

**Fig. 4:**
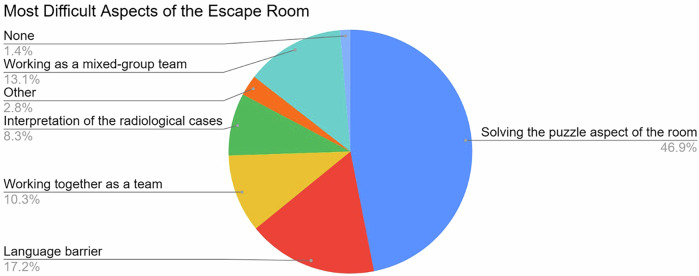
Rating of the most difficult aspect of the escape room

**Fig. 5:**
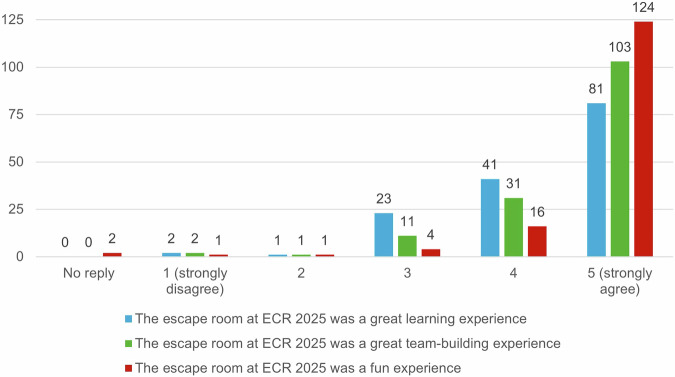
Survey results rating the learning experience (blue), the team-building experience (green), and the enjoyment (red) of the escape room

**Fig. 6:**
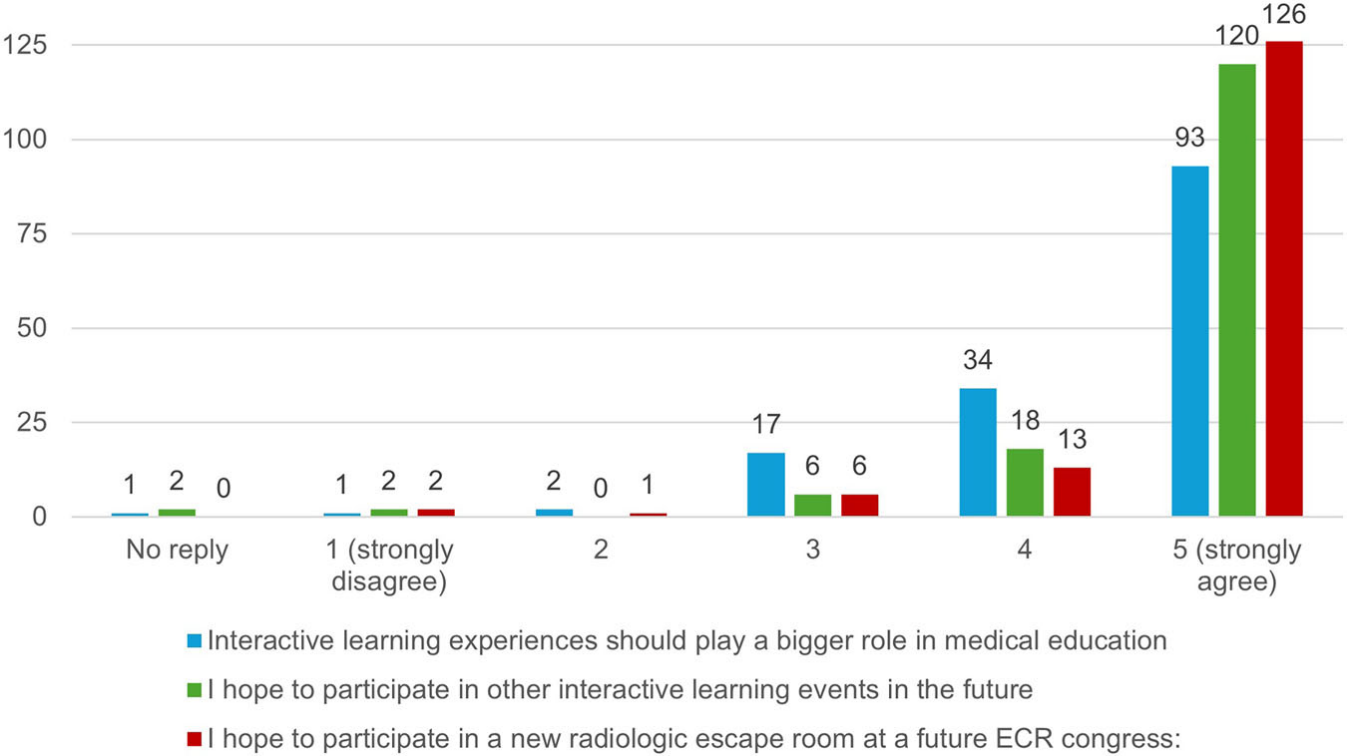
Survey results on the role of interactive experiences in medical education, discussing agreement that interactive learning experiences should play a larger role (blue), and the hope to participate in other interactive learning events (green) and in new radiologic escape rooms (red)

Although there was a relatively high rate of groups that did not complete the escape room in time, 84.2% rated the difficulty as just right. In the physician participants, 11 rated the room as too difficult (10.2%), and in the non-physician staff, 5 rated it as too difficult (12.5%). Almost half of the participants rated the puzzle aspect of the room as most difficult, with only 8.3% rating the radiological aspect as most difficult. About a quarter of participants rated teamwork as the largest problem in solving the room, either because of communication or being placed in a mixed-group (Fig. 4). Overall, participants strongly agreed with the statements that the escape room was a great learning, team-building, and fun experience, with the enjoyment factor being rated most positively, 84.9% strongly agreed with the statement. Generally, participants seemed to rate the team-building factor more positively than the learning effect. Full results are shown in Fig. 5. The agreement that interactive learning experiences should play a more significant role in medical education was high, with 86.4% agreeing. Further results are shown in Fig. 6.

## Discussion

While developing a radiology-themed escape room is time-intensive, with input needed from a large team of physicians, it results in an entertaining experience for residents, radiologists, and other non-physician staff. One of the most challenging aspects of designing the rooms is the inability to properly test the experience beforehand. During the first iterations, minor adjustments to the setup were necessary, technical issues had to be addressed, and certain puzzles simplified. Once these early problems were solved, the rooms operated smoothly. A few tips needed to be given to almost every group, with every staff member having slightly different approaches to the amount and complexity of the hints given. In certain instances, knowledge or language gaps needed to be bridged, e.g., in groups with non-physician staff, with the goal being to have these participants still be able to almost complete the escape room to avoid frustration. The escape rooms also included some practical tasks, such as a breast biopsy, which needed to be guided in some cases, if these were new to participants.

A variety of materials were necessary for each room design, many of which were reused each year. Most materials could be easily found on online marketplaces, where many low-cost solutions are available. The overall cost for these materials is estimated at 200€ for one room design. Objects for the puzzles, such as a UV-light (7€), locks (10€) or lock-boxes (20–30€), make up about half of the estimate, while the other half was spent on decorative items, including lab coats (15–20€), posters (5–20€) or more elaborate anatomic models (40–60€). Some specific models were bought directly from vendors, such as breast biopsy models (200€), while others were lent for educational purposes at little or no cost (FAST model, thrombectomy model).

Several other studies have looked at medical escape rooms. In particular, a radiology-themed room at the Radiological Society of North America congress in Chicago, USA, in 2018 showed a similar experience to this study, with the highest rating of overall enjoyment scored at 4.85/5, very similar to our average rating of 4.84/5. Participants in this hour-long escape room were solely radiology residents. Aspects of team-building and learning were also highlighted, and there was a preference for more interactive learning experiences [9]. A smaller, pediatric radiology-themed escape room for medical students from a team from London, UK, showed overall equal levels of enjoyment for the experience, with a proven learning experience in a pre- and post-experience exam [11]. Da Silva et al showed that group interactions and collective problem-solving can be favorable for knowledge retention, as “learning by doing” plays an important role in education [12]. Working in a team is crucial in clinical practice routines, and social learning helps create a healthy atmosphere, in which stressful situations can be tested [13, 14].

There are several limitations to our study. Emotional bias may be present, as participants filled out the exit survey after completing the room; participants being unable to finish in time may have less motivation to complete the survey, while possibly having a poorer view of the experience. Although the room was designed primarily for radiology residents, over 20% of survey respondents were non-physician staff. The room’s difficulty may not be adequate for this level and may have led to a more negative perception of the experience. The rooms were developed mainly by residents from Germany, relying heavily on their educational experience. Differences in residency programs between countries may lead to unexpected challenges in completing the radiological aspect of the puzzles. Additionally, the entire experience was developed in English, making it easier to solve puzzles for native-speaking countries.

Care was taken to compose mostly unbiased questions, however, some survey questions were inadvertently posed in a suggestive manner (i.e., “I hope to participate in other interactive learning events in the future,”) which may cause some further bias. While the survey results underlined the positive learning effect, we have no formal way of testing what was actually learned or retained, and the amount of newly acquired information may be small due to the limited scope of the escape room.

While a formal measurement of learning success (i.e., by pre- and post-experience quizzes) may be difficult to implement for an escape room without hinting at puzzles in the room or detracting from the overall enjoyment of the activity, a variety of skills may be practiced by this concept. The overall atmosphere with a time limit can simulate the real-life working environment, where time constraints are encountered daily. This aspect also forces participants to make quick diagnoses on presented images, as is often necessary in emergency settings in the hospital. The different puzzles that need to be solved simultaneously replicate the experience of working as a team to complete the daily workload. Some teams consisted of different medical professionals, such as radiologists and technologists, which may improve inter-professional teamwork. Future research should focus on developing methods for objective quantification of the learning effect of escape rooms, without detracting from the experience itself.

In summary, we have shown key steps to developing a radiology-themed escape room, many of which may be transferred to other settings. The experience provides a unique learning and team-building opportunity for medical staff. Participants rated it very positively, accompanied by a strong wish for further interactive learning opportunities.

## Supplementary information

Appendix1

Appendix2

## Data Availability

Full results of the survey data may be made available upon reasonable request.

## Abbreviations

ECR: European Congress of Radiology
ESR: European Society of Radiology
FAST: Focused Assessment with Sonography for Trauma
