# Supplementary material for: Escape rooms as an interactive learning experience: insights into designing a radiology-themed escape room and exit survey data
Source: Insights Imaging. 2025 Nov 1;16:240. doi: 10.1186/s13244-025-02127-x (PMC12579622; doi:10.1186/s13244-025-02127-x)
Supplement: Supplementary file 1 — Appendix1 [file 13244_2025_2127_MOESM1_ESM.docx]

Escape Room ECR 2025 Survey

Thank you for participating in our ECR 2025 Escape Room survey! Results may be published for research. All results are anonymous, your email address is not saved. If you wish to participate in the party ticket raﬄe, please enter your ESR-ID in the last ﬁeld.

# How much time was left on the clock (please enter '0' if you were unable to ﬁnish in time, leave blank if you do not remember)?

1. Which country are you from?

## Mark only one.

*[Dropdown list of nations of the world, not shown for clear arrangement & formatting ]*

# What gender do you identify as?

## Mark only one.


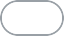
 Female
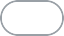
 Male


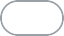
 Non-binary, transgender or any other
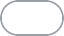
 Prefer not to answer

# How many members were in your team?

## Mark only one.


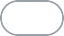
 2


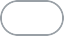
 3


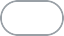
 4


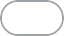
 5


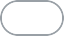
 (6)

# Were you part of a mixed team with members from different groups/institutions?

## Mark only one.


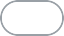
 No
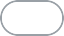
 Yes

# Was there any non-doctor staff in your escape room team?

## Mark only one.


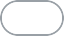
 No
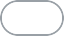
 Yes

# Was there anyone on your team with at least a board certiﬁcation in radiology or similar international standard (i.e. not a resident or student anymore)

## Mark only one.


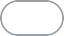
 No
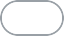
 Yes

# What best describes your level of training/role?

## Mark only one.


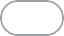
 Medical Student


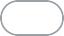
 Resident/Registrar/Physician in Training


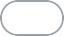
 < 10 years experience as Attending Physician/Consultant/Completed board certiﬁcation

> 10 years experience as Attending Physician/Consultant/Completed board certiﬁcation


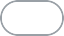
 Radiographer

Other:

# What best describes your practice setting?

## Mark only one.

University Hospital

Non-university tertiary care clinic Secondary care clinic/mid-size hospital Primary care clinic/small hospital Outpatient/private practice

Research only

Other:

# Have you played any non-radiologic/'regular' escape rooms previously?

## Mark only one.


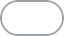
 No never
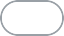
 Yes, 1-3 times


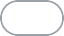
 Yes, more than 3 times

# Have you participated in any of the previous ECR Escape Rooms? (Select all that apply)

*Select all that apply.*


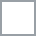
 At ECR 2019


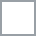
 At ECR 2023


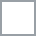
 At ECR 2024


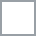
 "On Tour" 2023 Craiova, Romania
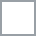
 "On Tour" 2023 Paris, France


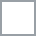
 "On Tour" 2024 Barcelona, Spain
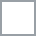
 "On Tour" 2024 Milan, Italy


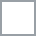
 "On Tour" 2024 Geneva, Switzerland
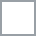
 "On Tour" 2024 Riga, Latvia


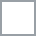
 "On Tour" 2024 Constanta, Romania

# Do you have a favorite version of the escape room?

## Mark only one.


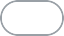
 ECR 2023 Room (Trauma Setting)


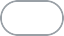
 ECR 2024 Room (Pulmonary Embolism Thrombectomy Setting)
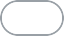
 ECR 2025 Room (Tumor Conference Setting)


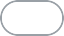
 I participated for the ﬁrst time this year

# How diﬃcult did you ﬁnd the escape room at ECR 2025?

## Mark only one.


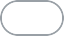
 Very diﬃcult


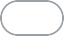
 Somewhat too diﬃcult
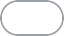
 Perfect diﬃculty


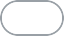
 Somewhat too easy
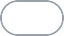
 Very easy

# Did you feel you received enough tips/help from the on-site team during the escape room at ECR 2025?

## Mark only one.


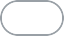
 Too little help was given


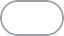
 Just the right amount of help was given
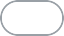
 Too much help was given

# The escape room at ECR 2025 was a great learning experience:

*Mark only one.*

1 2 3 4 5


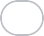

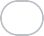

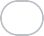

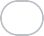

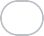


Stro Strongly agree

ngly

disag

ree

# The escape room at ECR 2025 was a great team-building experience:

*Mark only one.*

1 2 3 4 5


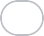

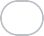

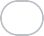

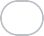

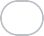


Stro Strongly agree

ngly

disag

ree

# The escape room at ECR 2025 was a fun experience:

*Mark only one.*

1 2 3 4 5


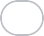

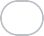

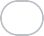

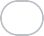

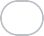


Stro Strongly agree

ngly

disag

ree

# What aspect of the escape room at ECR 2025 proved most challenging?

## Mark only one.


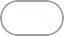
 Interpretation of the radiological cases
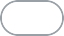
 Solving the puzzle aspect of the room


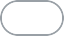
 Communicating and working together as a team


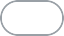
 Understanding of instructions, puzzles and other information due to language barrier


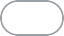
 Working together in a mixed-group team

Other:

# Interactive learning experiences should play a bigger role in medical education:

*Mark only one.*

1 2 3 4 5


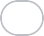

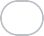

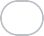

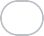

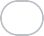


Stro Strongly agree

ngly

disag

ree

# I hope to participate in other interactive learning events in the future:

*Mark only one.*

1 2 3 4 5


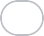

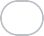

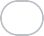

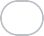

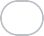


Stro Strongly agree

ngly

disag

ree

# I hope to participate in a new radiologic escape room at a future ECR congress:

*Mark only one.*

1 2 3 4 5


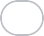

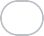

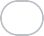

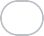

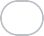


Stro Strongly agree

ngly

disag

ree

# Any other comments/feedback/ideas?

1. If you want to win ECR Party tickets, enter your ESR- personal ID below (6 digit number on your badge below the QR code):
