# Supplementary material for: Escape rooms as an interactive learning experience: insights into designing a radiology-themed escape room and exit survey data
Source: Insights Imaging. 2025 Nov 1;16:240. doi: 10.1186/s13244-025-02127-x (PMC12579622; doi:10.1186/s13244-025-02127-x)
Supplement: Supplementary file 2 — Appendix2 [file 13244_2025_2127_MOESM2_ESM.docx]

**Escape Room 2023 – Polytrauma:**


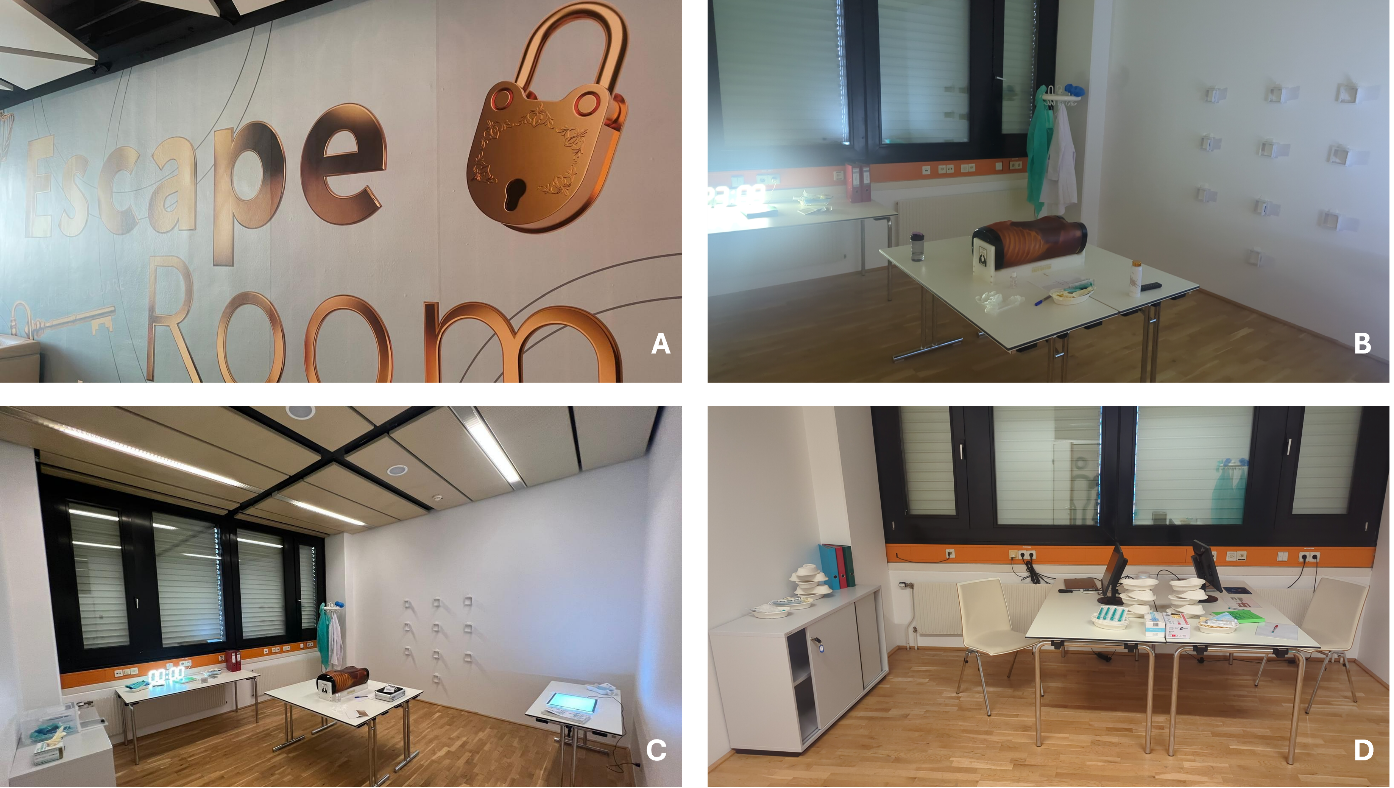


*A – Decorations in the lobby area; B – Set-up of the first room with Ultrasound phantom placed in the center, C – Set-up of the first room with a lightbox for X-Ray images and the revision doors on the wall, D – Set-up of the second room with two workstations for viewing images*

**Escape Room 2024 – Thrombectomy:**


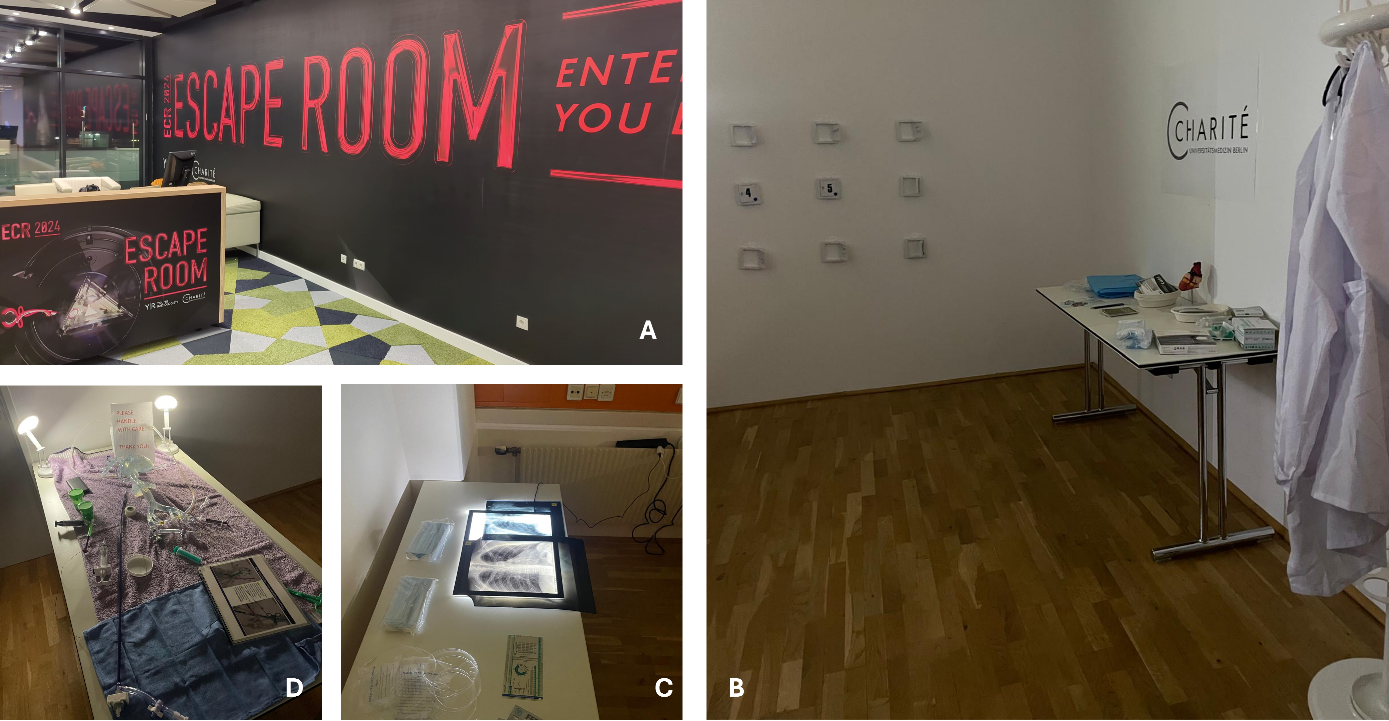


*A – Decorations in the lobby area, B – Set-up of the first room with revision doors on the wall, C – Set-up of the first room with lightbox for viewing X-Ray images, D – Set-up of the second room with the thrombectomy model*

**Escape Room 2025 – Tumor Conference**


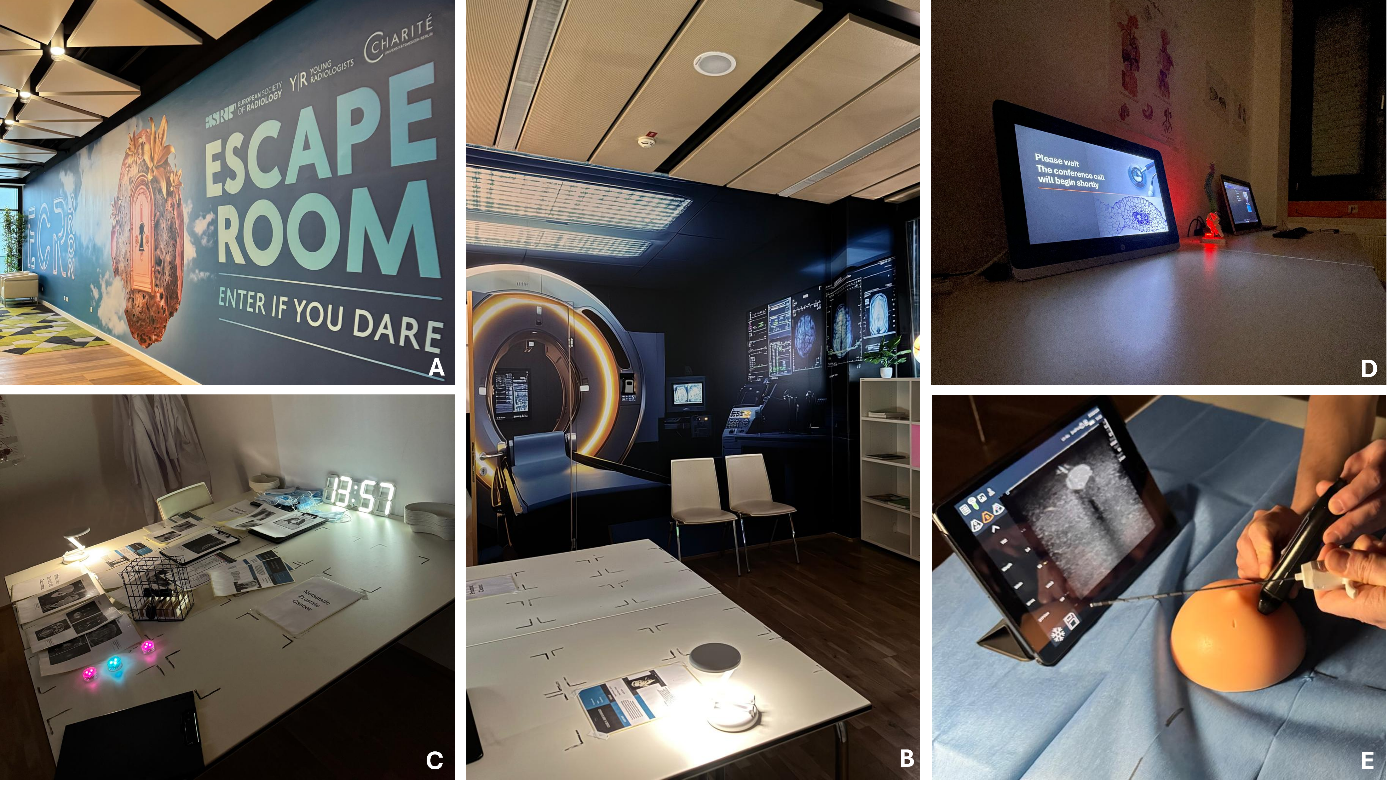


*A – Decorations in the lobby area, B - Set-up of the first room with table for sorting cases, C – close up image of the grid for sorting patient cases with timer, D – Set-up of the second room with workstation for viewing staging exams and screen for showing completion video, E – close up of breast biopsy*
